# Direct Molecular Detection and Genotyping of *Borrelia burgdorferi* from Whole Blood of Patients with Early Lyme Disease

**DOI:** 10.1371/journal.pone.0036825

**Published:** 2012-05-08

**Authors:** Mark W. Eshoo, Christopher C. Crowder, Alison W. Rebman, Megan A. Rounds, Heather E. Matthews, John M. Picuri, Mark J. Soloski, David J. Ecker, Steven E. Schutzer, John N. Aucott

**Affiliations:** 1 Ibis Biosciences Inc., an Abbott Company, Carlsbad, California, United States of America; 2 Divisions of Rheumatology and Internal Medicine, Department of Medicine, Johns Hopkins School of Medicine, Baltimore, Maryland, United States of America; 3 Department of Medicine, University of Medicine and Dentistry of New Jersey-New Jersey Medical School, Newark, New Jersey, United States of America; 4 Lyme Disease Research Foundation, Lutherville, Maryland, United States of America; Naval Research Laboratory, United States of America

## Abstract

Direct molecular tests in blood for early Lyme disease can be insensitive due to low amount of circulating *Borrelia burgdorferi* DNA. To address this challenge, we have developed a sensitive strategy to both detect and genotype *B. burgdorferi* directly from whole blood collected during the initial patient visit. This strategy improved sensitivity by employing 1.25 mL of whole blood, a novel pre-enrichment of the entire specimen extract for *Borrelia* DNA prior to a multi-locus PCR and electrospray ionization mass spectrometry detection assay. We evaluated the assay on blood collected at the initial presentation from 21 endemic area patients who had both physician-diagnosed erythema migrans (EM) and positive two-tiered serology either at the initial visit or at a follow-up visit after three weeks of antibiotic therapy. Results of this DNA analysis showed detection of *B. burgdorferi* in 13 of 21 patients (62%). In most cases the new assay also provided the *B. burgdorferi* genotype. The combined results of our direct detection assay with initial physician visit serology resulted in the detection of early Lyme disease in 19 of 21 (90%) of patients at the initial visit. In 5 of 21 cases we demonstrate the ability to detect *B. burgdorferi* in early Lyme disease directly from whole blood specimens prior to seroconversion.

## Introduction

Lyme disease, caused by the tick-borne bacteria *Borrelia burgdorferi*, is the most commonly reported vector-borne infectious disease in North America. The number of yearly cases reported to the CDC has steadily increased since 1982 when case reporting began, with 20,000–30,000 cases now reported each year [1], [2].

If promptly diagnosed and correctly treated, outcomes for early Lyme disease are generally considered to be excellent. Direct *Borrelia* molecular tests, such as PCR, from blood can detect and identify active infection sooner than serologic tests but typically these tests have suffered from low assay sensitivity for clinical use. For example early studies using PCR to detect *Borrelia* in the blood during active infection had limited success with sensitivities of only 18.4% and 26.1% [3], [4]. Studies in recent years have reported a higher detection rate down to 10 genome copies through sampling larger blood volumes, in some cases culturing prior to PCR, and by using different PCR techniques such as qPCR or nested PCR [5], [6], [7].

In the past, PCR assays for Lyme disease did not have the benefit of current advances in sample preparation [8] or target DNA amplification techniques that could enable the detection of single digit copy numbers of *Borrelia* DNA in large volume samples. Studies have shown PCR detection of *B. burgdorferi* can be enhanced by culturing the specimen in growth medium prior to PCR indicating the presence of organism below the limits of detection by PCR [6].

We have previously applied broad-range PCR and electrospray ionization mass spectrometry (PCR/ESI-MS) for the detection of vector-borne pathogens such as *Ehrlichia,* Powassan virus, and canine heartworm [9], [10], [11]. The basis of this detection and identification is a multi-locus broad range PCR followed by the determination of the mass of the amplicons using automated electrospray ionization mass spectrometry. From the masses of the amplicons, the numbers of DNA base pairs A's, G's, C's and T's in each amplicon are determined. By analysis of the base compositions of amplicons from all primer pairs, the organisms present in the sample can be identified from a database of all known base count signatures and quantified [12], [13]. This technique has the advantage of rapidly identifying pathogens, genotyping pathogens and can identify new genetic variants. To address the need for a better test for early Lyme disease we have improved the sensitivity of PCR/ESI-MS by the use of 1.25 mL of whole blood and an isothermal amplification (IA) for *Borrelia* DNA on the entire specimen extract prior to a multi-locus PCR/ESI-MS assay.

## Methods

### Ethics Statement

The study was approved by the Johns Hopkins Medicine Institutional Review Board. Written informed consent was received from participants prior to inclusion in the study.

**Figure 1:**
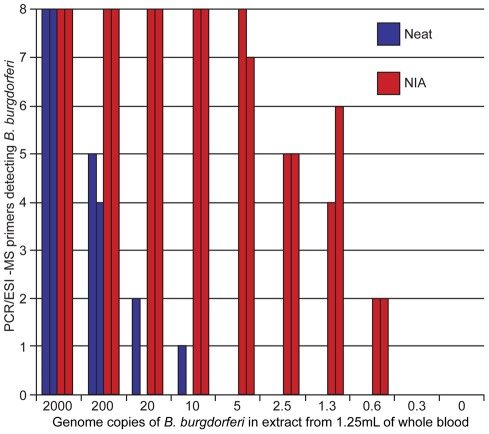
*B. burgdorferi* detections with untreated and IA treated whole blood extracts spiked with *B. burgdorferi* genomic DNA. Nucleic acid extracts from 1.25 mL of whole blood were spiked with decreasing of amounts of *B. burgdorferi* genomic DNA. In duplicate, specimens were either untreated or underwent IA before use in the PCR/ESI-MS *B. burgdorferi* detection/genotyping assay. The number of primer pairs in the assay detecting *B. burgdorferi* DNA was recorded for each specimen.

### Early Lyme disease patient specimens

A set of 29 whole blood samples including pretreatment (acute) and post-treatment (convalescent) samples were obtained and analyzed from consecutively enrolled adult patients from an area endemic for Lyme disease (Baltimore and adjacent counties of Maryland) and not know to be endemic for STARI. Patients presented in the spring and summer of 2009 with a rash consistent with EM and systemic symptoms consistent with an acute *B. burgdorferi* infection such as general malaise, headache, and the ‘flu-like’ symptoms. These patients had been referred to a primary care physician with infectious disease training (JA) and previous experience identifying EM in research settings [14]. The initial samples were obtained prior to initiating a three week course of doxycycline antibiotic therapy. Patients with a prior history of Lyme disease or symptom duration of their current illness of longer than 3 months or a history of chronic illnesses associated with immune suppression or chronic functional somatic disorders were excluded. To ensure our sample cohort was infected with *B. burgdorferi* and did not just present with an EM-like rash due to other causes, all patients, in addition to a clinical diagnosis, were required to have laboratory evidence of infection by 2-tiered serological testing following CDC testing guidelines for acute and convalescent testing [15]. Of the 29 initial patients with EM and clinical symptoms, 21 also had serologic evidence of exposure to *B. burgdorferi* infection and are the focus of this report.

Endemic area negative control specimens were procured from Biomed Supply Inc. (Carlsbad, CA). Paired whole blood and serum specimens were collected December 2010 from a total of 44 healthy donors at a donation site in Pennsylvania for the molecular and serological testing.

### Serological and other analyses

At the initial, pre-treatment study visit 2-tiered antibody testing for *B. burgdorferi* were performed on the patient's serum sample as part of the patient clinical evaluation. Patients with negative 2-tiered results were tested again following the three week antibiotic treatment period in a follow-up study visit. All serologic testing for both the patient specimens and the negative controls was performed through the same commercial laboratory using standardized protocols. Results were interpreted according to CDC reccommendations for test interpretation for Lyme disease [15].

### DNA extraction from blood specimens

A combination of bead-beating and magnetic bead isolation was used to extract nucleic acids from 1.25 mL of whole EDTA blood. The blood was mixed in 2.0 mL screw-cap tubes (Sarstedt, Newton NC) filled with 1.35 g of 0.1 mm yttria-stabilized zirconium oxide beads (Glen Mills, Clifton, NJ), 25 µL Proteinase K solution (Qiagen, Valencia, CA), 142 µL of 20% SDS solution (Ambion, Austin, TX) and 1 µL of DNA extraction control (Abbott Molecular, Des Plains, IL). The mixture was then homogenized in a Precellys 24 tissue homogenizer (Bioamerica Inc., Miami, FL) at 6,200 rpm for 3 sets of 90 sec with 5 sec between sets. The homogenized lysates were then incubated at 56°C for 15 min and then centrifuged for 3 min at 16,000 g in a bench top microcentrifuge. To isolate nucleic acids 1 mL of the supernatant was transferred to a 24-well deep-well Kingfisher plate (Thermo Scientific, Waltham, MA) along with 1.1 mL of Abbott lysis buffer (Abbott Molecular), and 160 µL of magnetic particles (Abbott Molecular). The plate was then used on a Kingfisher Flex (Thermo Scientific) to automate binding, washing, and elution of the nucleic acids. The specimens were incubated for 16.5 minutes in the lysis buffer at 56°C. Specimens were then washed once in Wash buffer I (Abbott Molecular), and three times in Wash buffer II (Abbott Molecular), with 1 min incubation for each wash step. The magnetic beads were then dried for 3 min at 65°C, and nucleic acids were eluted into 250 µL of elution buffer (Abbott Molecular), by incubating the magnetic particles at 65°C for 3 min.

### Isothermal amplification of *B. burgdorferi* target DNA

Each of the seven target loci were amplified using 50 oligonucleotide primers flanking the locus (**Table S1**). The primers were purchased in 96 well plates (IDT DNA, Ames, IA) at a 1 mM conc and mixed in equal proportions to create a 1 mM primer pool in tris EDTA buffer. Primers were designed using *B. burgdorferi* B31 genome sequence (gi|15594346) with a GC content of 25 to 50%, spaced 6–10 nucleotides apart with a target TM of 52°C. The pooled primers were dialyzed twice for 4 hours at 4°C against a 4L solution containing 10 mM Tris pH 8.0 and 50 µM EDTA pH 8.0 using a 5 mL Float-A-Lyzer G2 with a 0.5–1 kDa MW cutoff (Spectrum Laboratories, Rancho Dominguez, CA). Multiple primer isothermal amplification (IA) was performed in a in a 0.6 mL PCR tube (Axygen Inc., Union City, California) containing 200 µL of nucleic acid extract, 22.5 µl Ibis 10X PCR Buffer II (Ibis Biosciences, Carlsbad, CA) [13], 0.2 µM dNTPs (Bioline, Tauton, MA), and 10 µM oligo mix. The reactions were incubated at 95°C for 10 min followed by a cooling to 56°C in a MJ Thermocycler (Bio-Rad Laboratories, Hercules, CA.). 11.25 U of Bst DNA polymerase, Exonuclease minus (Lucigen, Middleton, WI) enzyme was added to the reactions and they were incubated at 56°C for 2 hours followed by an 80°C heat inactivation for 20 min. The resulting reaction was used directly in the subsequent PCR without purification.

### Molecular detection and genotyping of *B. burgdorferi* from whole blood from patients with clinically diagnosed early Lyme disease

*Borrelia* was detected and genotyped by processing 2 µL of *Borrelia* enriched nucleic acid extracts per PCR reaction on a previously described broad-range PCR/ESI-MS *Borrelia* genotyping assay that targets eight different loci in the *Borrelia* genome [16]. Amplicons were analyzed using the research use only PLEX-ID system (Abbott, Des Plaines, IL). When all eight primers produced amplicons, a genotype of the *Borrelia* was determined as previous described [16]. Detection of a *Borrelia* amplicon from one or more of the eight primer pairs in the assay was used to positively identify *Borrelia* DNA in a specimen.

### Statistical analysis

The 95% confidence interval of our PCR positive blood specimens undergoing IA/PCR/ESI-MS was calculated using the modified Wald method which is useful when calculating from a small sample size [17]. This method was also used on calculating the 95% confidence interval for the combined assay approach. Comparison of the specimens that were treated with IA verses no treatment was performed using the McNemar's exact test using R for the analysis (www.r-project.org).

## Results

### Early Lyme disease patients

Of the 29 patients with clinically diagnosed EM, 21 also had laboratory evidence of *B. burgdorferi* exposure by a positive two-tiered serology test either on the initial physician visit or the follow-up visit three weeks later. These 21 patients were included in the study cohort and downstream analysis. (**Table S2**).

### Improved PCR/ESI-MS sensitivity by Isothermal Amplification

To detect the low levels of *Borrelia* genomic DNA in whole blood samples from early Lyme disease patients we developed an isothermal amplification (IA) assay that amplifies the seven *B. burgdorferi* target regions used with a previously described *Borrelia* broad-range PCR electrospray ionization mass spectrometry detection and genotyping assay (PCR/ESI-MS)[16]. The strategy behind this approach uses a combination of a strand-displacing DNA polymerase in conjunction with multiple primers to selectively amplify the target region in an isothemeral reaction. The use of multiple primers flanking the target sequence allows outer primers to displace the DNA strands created by inner primers allowing the newly displaced strand to function as a template for further amplification in a manner similar to multiple displacement amplification [18]. The IA assay was designed to specifically work with the entire output of DNA extraction from 1.25 mL of whole EDTA blood to ensure amplification of low levels of *Borrelia* DNA that may be present in the specimens. The *Borrelia* enriched DNA can then be used directly in the PCR/ESI-MS assay without any purification. Since the IA is coupled with PCR/ESI-MS specificity for the target detection is maintained through the PCR/ESI-MS. Using extracts from 1.25 mL of human blood spiked with various amount of quantified *B. burgdorferi* DNA (Barbara J. Johnson CDC, Fort Collins, CO) (200 genome copies down to 0.3 genome copies) we compared the sensitivity of the PCR/ESI-MS assay to detect *B. burgdorferi* with and without the IA *B. burgdorferi* DNA enrichment (**Figure 1**). Detection was determined when one or more of the eight primers detected *B. burgdorferi*. As shown in **Figure 1**, samples that were not enriched (neat) reproducibly detected down to 200 *B. burgdorferi* genomes. When the IA *B. burgdorferi* DNA enrichment was employed the sensitivity was reproducibly improved down to 0.6 genomes. This demonstrated the IA *B. burgdorferi* DNA enrichment process resulted in over a 200-fold increase in sensitivity in the PCR compared to untreated.

The ability of IA to increase detection sensitivity in actual blood specimens from patients was also evaluated. Blood extract from the 21 early Lyme disease patients was extracted in duplicate. One replicate underwent IA *B. burgdorferi* DNA enrichment prior to PCR/ESI-MS while the other remained untreated. Each replicate was then run on the PCR/ESI-MS assay. In the untreated samples we detected *B. burgdorferi* in two of the 21 patients. In the IA *B. burgdorferi* DNA enriched samples we detected *B. burgdorferi* in 13 of the patient samples. When comparing samples from the same patient that were processed with and without IA using the McNemar's exact test, IA provided a significantly higher rate of detection (P = 0.001). This finding demonstrates that the use of our isothermal amplification prior to PCR can significantly improve the sensitivity of the PCR based assays.

### Molecular detection of *Borrelia burgdorferi* from whole blood by isothermal amplification, PCR/ESI-MS, and two-tiered serology

The IA/PCR/ESI-MS assay detected *B. burgdorferi* in 13 of 21 patient specimens (62% with a 95% confidence interval of 40%-79%) while two-tiered serology was positive in 14 of the 21 specimens (67% with a 95% confidence interval of 45%-83%) at the initial diagnostic pre-treatment visit **Table 1**. Of the 44 control patient sera, a single sample was seropositive by ELISA and IgG western blot, but all control specimens were IA/PCR/ESI-MS negative (**Table 1**). This low rate in seropositivity in our sample set from the eastern United States is consistent with the known background seroreactivity expected from remote exposure in an endemic region [19], [20].

**Table 1:** Comparison of isothermal amplification PCR/ESI-MS assay and two-tier serology for the detection of early Lyme disease on initial patient visit.

		Early two-tier Serology		
		Pos	Neg	Total
Early Lyme Disease	IA/PCR/ESI-MS +	8	5	13
	IA/PCR/ESI-MS -	6	2	8
	Total	14	7	21
Control group	IA/PCR/ESI-MS +	0	0	0
	IA/PCR/ESI-MS -	1	43	44
	Total	1	43	44

Of the 13 early Lyme disease patients that tested positive by the IA/PCR/ESI-MS assay, 5 were negative by the 2-tiered test at the first physician visit (**Table 2**). the 8 patients negative by the IA/PCR/ESI-MS, 6 were positive by serology on initial visit and the remaining 2 where found positive by serology on the follow-up physician study visit (**Table 2**).

**Table 2:** Specimen results for IA/PCR/ESI-MS, initial, and follow-up two-tiered serology testing.

# patients	# with Muliple EM	IA/PCR/ESI-MS	Early two-tier serology	Follow-up two-tier serology	Genotype Found
8	6	Pos	Pos	ND	6, 6, 7, 22, 33
5	1	Pos	Neg	Pos	1, 10, 76
6	4	Neg	Pos	ND	ND
2	0	Neg	Neg	Pos	ND

Examining the ability to detect *B. burgdorferi* infection in patients with early Lyme disease at the initial physician visit with either laboratory test, IA/PCR/ESI-MS or two-tiered serology, resulted in 19 of 21 patient specimens (90% with a 95% confidence interval of 70% to 99%) having a positive detection of *B. burgdorferi* infection.

### Genotyping of *B. burgdorferi* from patients with early Lyme

The eight primer pairs used in the IA/PCR/ESI-MS assay have been previously used to genotype *B. burgdorferi* [16]. A combination of the basecount signatures from the 8 loci can provide genotyping of *B. burgdorferi* at a similar if not higher level of resolution compared to ospC typing. Of the 14 IA/PCR/ESI-MS positive specimens 9 specimens detected *B. burgdorferi* with all eight primers thus producing a genotype [16]. Of these specimens two were positive with genotype 6 and two specimens were positive with genotype 7 [16]. Four specimens were positive with genotypes 1, 10, 22 and 33. One specimen produced a previously unseen genotype and was designated genotype 76 (**Table 2**). This new genotype is similar to the previously seen genotype 14 except the basecount signatures for primer pairs BCT3516 was A38G28C24T38 and BCT3518 was A38G22C14T36.

## Discussion

Our results demonstrate an improved strategy for detecting *B. burgdorferi* infection from whole blood in patients with erythema migrans and acute Lyme disease. Employing isothermal amplification of DNA prior to PCR, we were able to detect the low numbers of organisms often associated with blood stream invasion in early Lyme disease. For this initial study the choice of patients with early untreated Lyme disease was important because of the known relationship of EM with acute infection and the published rates of bacteremia in this setting using *Borrelia* culture [21]. The ability to confirm early infection with direct pathogen based tests has significant research applications and the potential future clinical application of DNA based diagnosis.

The development of pathogen based diagnostic tests in Lyme disease is important because the only objective clinical finding that can be used to diagnose Lyme disease, the EM, is subjective in interpretation and can result in false positive and false negative diagnosis [22]. The identification of EM is further limited by the variability in the appearance of the skin lesion creating the potential for clinical misdiagnosis [23]. The most common current laboratory tests for Lyme disease, two-teired serology, can also provide false positive or negative results due to variations in the immune response of the patient. Because of the biologically delayed antibody response in the initial 3 weeks of infection, the sensitivity of serological tests may be as low as 40% [24], [25]. False positive tests can occur with IgM immunoblots and a positive IgG immunoblot cannot distinguish active infection from past exposure.

Since the adoption of the current two-tier serologic based test for Lyme disease surveillance there have been few practical alternative diagnostic tests available to the clinician. The ability to culture *B. burgdorferi* has been important for use in research applications to confirm the diagnosis of *B. burgdorferi* infection, but is not available or practical for use in a clinical setting because it requires weeks before cultures become positive. Past attempts at PCR diagnosis for North American Lyme disease with blood samples has been largely unsuccessful due to the low sensitivity [3], [4]. European researchers have reported varying rates of success using PCR to detect members of the *B. burgdorferi* s.l. group from blood or its components; 7.5% up to 78.1% [26], [27], [28]. The difficulty of detecting Lyme *Borrelia* in the blood has been attributed to the low numbers of organisms circulating in the blood stream in acute infection [7], [14], [29]. In recent studies in North America, using larger blood volumes in addition to utilizing more sensitive nested PCR tests have had increased success in detecting the low densities of *Borrelia* that are typically present in the blood in early Lyme disease [6], [7]. These studies also demonstrated that a direct detection strategy can work provided enough blood is examined and the assay is sensitive down to genome copies numbers in the 10–100 range.

To overcome the challenge of extremely low density of spirochetes in the blood and increase assay sensitivity we utilized a combination of strategies. By using a relatively large volume of whole blood we increased the probability of *B. burgdorferi* DNA being present in the specimen. Next, the IA strategy developed here enriches the entire nucleic acid extract for *Borrelia* DNA prior to PCR thereby increasing the probability that any *Borrelia* DNA present is detected. Lastly we used a mass spectrometry-based multi-locus assay to increase the number of possible targets in the *Borrelia* genome we may detect. Extracts subjected to IA can also be used to screen for other pathogens without affecting the sensitivity of the individual assays (data not shown). All of the specimens in the study were tested for other tick-borne pathogens including *Babesia*, *Anaplasma, Ehrlichia* and no co-infections were found (data not shown). A key technical development of the isothermal amplification is that it uses the entire output of a whole blood DNA extraction and employs a set of 50 primers per target locus (350 primers total) to assure amplification of the *B. burgdorferi* target loci even the presence of high levels of human DNA. The isothermal amplification is a relatively simple addition to a PCR assay work flow and this approach could be applied to other assays where target DNA of interest may be present but at levels below the limits of detection by PCR. Furthermore the method should be applicable to DNA extracts from a variety of specimen types such as tissue, cerebrospinal fluid, serum, plasma or synovial fluid.

The IA approach combined with the *Borrelia* PCR/ESI-MS genotyping assay not only has the ability to detect extremely low genome copies of *B. burgdorferi* in a specimen, it is also able to identify the specific genotype of the infecting *B. burgdorferi*. When the IA/PCR/ESI-MS assay detects *Borrelia* with all eight primer pairs a multi-locus genotype can be determined [16]. From our set of 13 positive specimens 9 produced a full genotype representing seven unique genotypes (genotypes 1, 6, 7, 10, 22, 33 and 76) [16]. Earlier studies have indicated some strains may be associated with a propensity to disseminate and perhaps to specific organ systems [30]. Had we been able to perform skin biopsies in the subjects, we may have been able to ascertain which blood PCR negative subjects were skin PCR positive and had a genotype that appeared to be skin-restricted. Future studies which examine both skin biopsies and blood in patients with early Lyme disease could examine which infecting *Borrelia* genotypes might be restricted to the skin and which genotypes have the ability to disseminate into the blood stream and other tissues.

Comparison of IA/PCR/ESI-MS and 2-tier serologic testing showed that the IA/PCR/ESI-MS based method was able to identify patients early in the course of infection at a time when many had not yet developed a diagnostic humoral immune response. Five of the IA/PCR/ESI-MS positive specimens were negative by the 2-tier test on the initial physician visit. When retested at visit two, 3 weeks later, all had evidence of seroconversion indicating acute exposure. Results of this analysis showed detection of early Lyme disease in 13 patients (62%). In one of the 8 patients excluded from the cohort due to not having a positive two-tiered serology test that met CDC guidelines we detected *B. burgdorferi* DNA with the IA/PCR/ESI-MS assay (specimen # 9, **Table S2**). While this patient was not part of the 21 patients in the cohort due to negative serology test results, this sample did show increased sero-reactivelty at the follow-up two-tier test and most likely represented a *Borrelia* positive patient with a weak serologic response. The combined results of our direct detection assay with initial physician visit serology resulted in the detection of 19 of 21 (90%) of patients at the initial visit.

The IA/PCR/ESI-MS assay can be performed in 8 hours and can detect *B. burgdorferi* in early Lyme disease prior to seroconversion, indicating the presence of an active infection. This distinguishes our assay from traditional serological tests, which cannot differentiate active infection from prior exposures. A recent study examining paired samples of skin biopsies and blood found that of 249 patients with positive skin biopsy cultures only 108 (43.4%) had positive blood cultures [31]. This is similar to our results detecting *B. burgdorferi* in the blood of 62% of patients with an EM rash and could point to a limitation based upon the true biologic rates of bacteremia with early *B. burgdorferi* infection and not assay sensitivity [21]. When positive, the IA/PCR/ESI-MS assay can provide an immediate early and unambiguous result in a single test without the need for a later second visit convalescent sample as with serology. Additionally the genotype information provided may give insight into the pathogenicity or biologic behavior of the infecting strain that can be explored in larger studies. The application of our IA/PCR/ESI-MS assay for use in specific clinical settings warrants further study with defined diagnostic criteria to assess its clinical applicability [32]. One such example would be a study in high risk patients with “summer flu-like” illness in endemic areas for Lyme disease. Many other blood-borne infections may contain low levels of circulating pathogen DNA and would also benefit from the IA approach to improve PCR sensitivity.

## Supporting Information

Table S1 *Borrelia* isothermal amplification primers. Primer sequences used during the isothermal amplification of *Borrelia* DNA for each of the seven regions targeted.
(XLSX)

Table S2 Genotypic, and serological analysis of specimens by IA/PCR/ESI-MS. Results of PCR, IA/PCR, and 2-tier serology by patient ID. If IA/PCR was positive the number of primers detecting is given in parenthesis. ND  =  Not determined; NA  =  Not applicable; IC  =  Incomplete genotype signature; Control samples are prefixed with “C”.
(XLSX)
